# Transcatheter and surgical aortic valve replacement in patients with bicuspid aortic valve

**DOI:** 10.1007/s00392-020-01761-3

**Published:** 2020-10-24

**Authors:** Annastiina Husso, Juhani Airaksinen, Tatu Juvonen, Mika Laine, Sebastian Dahlbacka, Marko Virtanen, Matti Niemelä, Timo Mäkikallio, Mikko Savontaus, Markku Eskola, Peter Raivio, Antti Valtola, Fausto Biancari

**Affiliations:** 1 Heart Center, Kuopio University Hospital, Kuopio, Finland; 2 Heart Center, Turku University Hospital, and University of Turku, Turku, Finland; 3 Heart and Lung Center, Helsinki University Hospital, Haartmaninkatu 4, P.O. Box 340, 00029 Helsinki, Finland; 4 Research Unit of Surgery, Anesthesiology and Critical Care, University of Oulu, Oulu, Finland; 5 Heart Hospital, Tampere University Hospital and Faculty of Medicine and Health Technology, Tampere University, Tampere, Finland; 6 Department of Internal Medicine, Oulu University Hospital, Oulu, Finland

**Keywords:** Bicuspid aortic valve, Aortic stenosis, TAVR, TAVI, Transcatheter, Aortic valve replacement

## Abstract

**Objectives:**

To compare the outcomes after surgical (SAVR) and transcatheter aortic valve replacement (TAVR) for severe stenosis of bicuspid aortic valve (BAV).

**Methods:**

We evaluated the early and mid-term outcome of patients with stenotic BAV who underwent SAVR or TAVR for aortic stenosis from the nationwide FinnValve registry.

**Results:**

The FinnValve registry included 6463 AS patients and 1023 (15.8%) of them had BAV. SAVR was performed in 920 patients and TAVR in 103 patients with BAV. In the overall series, device success after TAVR was comparable to SAVR (94.2% vs. 97.1%, *p* = 0.115). TAVR was associated with increased rate of mild-to-severe paravalvular regurgitation (PVR) (19.4% vs. 7.9%, *p* < 0.0001) and of moderate-to-severe PVR (2.9% vs. 0.7%, *p* = 0.053). When newer-generation TAVR devices were evaluated, mild-to-severe PVR (11.9% vs. 7.9%, *p* = 0.223) and moderate-to-severe PVR (0% vs. 0.7%, *p* = 1.000) were comparable to SAVR. Type 1 N-L and type 2 L-R/R-N were the BAV morphologies with higher incidence of mild-to-severe PVR (37.5% and 100%, adjusted for new-generation prostheses *p* = 0.025) compared to other types of BAVs. Among 75 propensity score-matched cohorts, 30-day mortality was 1.3% after TAVR and 5.3% after SAVR (*p* = 0.375), and 2-year mortality was 9.7% after TAVR and 18.7% after SAVR (*p* = 0.268)

**Conclusions:**

In patients with stenotic BAV, TAVR seems to achieve early and mid-term results comparable to SAVR. Type 1 N-L and type 2 L-R/R-N BAV morphologies had higher incidence of PVR. Larger studies evaluating different phenotypes of BAV are needed to confirm these findings.

**Clinical trial registration:**

ClinicalTrials.gov Identifier: NCT03385915.

**Graphic abstract:**

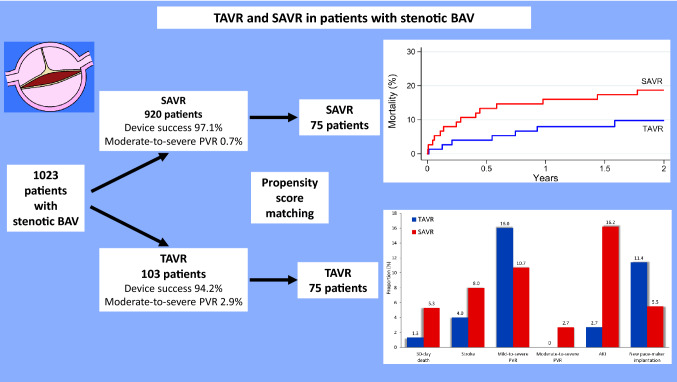

## Introduction

Bicuspid aortic valve (BAV) is the most common congenital cardiac defect with a prevalence of 0.65% in the most recent autopsy series [1]. It has been speculated that the abnormal shear stress caused by altered morphology of the aortic valve may over time lead to leaflet calcification [2]. In fact, aortic valve stenosis has been observed in 12–37% of patients with BAV, and this typically occurs at younger age than in subjects with tricuspid aortic valve [3]. Compared to tricuspid aortic valves, stenotic BAVs are more likely to have heavily calcified leaflets and raphes as well as dilatation of the aortic annulus and root [4]. Recent randomized studies showed comparable outcomes in tricuspid AS after TAVR and SAVR even in low-risk patients [5, 6]. However, patients with BAV were excluded from these clinical trials, due to concerns about technical issues related to its morphological features and the possible associated aortopathy. Indeed, the increased risk of vascular complications, permanent pacemaker implantation and paravalvular regurgitation (PVR) after TAVR in BAV is a matter of concern [7, 8]. The risk of such complications seems to be reduced with the use of newer-generation TAVR devices [7, 9, 10], but current guidelines do not provide any advice regarding the routine use of TAVR in BAV patients [11, 12]. TAVR in BAV is performed in patients with contraindications for SAVR [13], with some evidence of similar device success to tricuspid aortic valves [8, 14]. Still, there are no comparative data on TAVR and SAVR in patients with stenotic BAV. In this study, we sought to analyze the early and mid-term outcomes of these treatment methods in patients with BAV from a nationwide registry.

## Methods

### Study population

The nationwide FinnValve registry (ClinicalTrials.gov identifier NCT03385915) included data on unselected, consecutive patients who underwent TAVR or SAVR with a bioprosthesis for AS with or without coronary artery revascularization at all five Finnish University hospitals (Helsinki, Kuopio, Oulu, Tampere, and Turku University Hospitals) from January 2008 to November 2017. This study was approved from the institutional research boards of each participating center.

Exclusion criteria for this registry were: age < 18 years, previous surgical or transcatheter intervention on the aortic valve, acute endocarditis, isolated aortic valve regurgitation, or other major concomitant surgical procedures on other heart valves or thoracic aorta. Data were retrospectively collected from patient’s electronic records as well as from electronic laboratory and transfusion service databases. Data on mortality and repeat procedures on the aortic valve were retrieved from the electronic registry of the Finnish Institute for Health and Welfare. Follow-up was considered complete for all patients residing in Finland, while follow-up was truncated at the hospital discharge for those few patients residing abroad.

Patients with BAV were the subjects of this study. Data on the nature of the aortic valve were retrieved solely from the operative report of patients who underwent SAVR because these patients did not undergo routinely preoperative aortic computed tomography. Data on different morphological patterns of BAV of patients who underwent TAVR were retrospectively classified according to the Sievers–Schmidtke’s classification [15] based on the findings of preoperative aortic computed tomography reviewed by experienced on-site investigators.

Herein, we considered newer-generation TAVR prostheses: the Sapien 3 (Edwards Lifesciences, Irvine, CA, USA), Lotus (Boston Scientific, Marlborough, MA, USA), Acurate Neo (Boston Scientific, Marlborough, MA, USA) and CoreValve Evolut devices (Medtronic, Minneapolis, MN, USA); and older-generation devices: the CoreValve (Medtronic, Minneapolis, MN, USA) and Sapien XT (Edwards Lifesciences, Irvine, CA, USA) devices.

### Outcomes

The primary outcomes of this study were 30-day and 2-year all-cause mortality as well as PVR. The secondary outcomes were device success, stroke, conversion to cardiac surgery, prosthesis migration, coronary ostium occlusion, aortic dissection or rupture, major vascular complications, red blood transfusion, reoperation for intrathoracic or peripheral bleeding, acute kidney injury, new renal replacement therapy, deep sternal wound infection or mediastinitis, atrial fibrillation and new permanent pacemaker implantation and hospital stay. The secondary outcomes were defined as occurring during the index hospitalization. PVR was estimated by transthoracic echocardiography before discharge. Device success was defined as the absence of 30-day mortality, correct positioning of a single prosthetic heart valve into the proper anatomical location, and no moderate or severe PVR. Stroke and major vascular complications were defined according to the Valve Academic Research Consortium 2 (VARC-2) criteria [16]. Severe bleeding was defined according to the European Coronary Artery Bypass Grafting (E-CABG) bleeding scores 2–3, i.e., transfusion of more than 4 units of red blood cells and/or reoperation for mediastinal and/or peripheral bleeding [17]. In this study, the VARC-2 definition of major and life-threatening bleeding was not applied because, unlike patients undergoing TAVR, a significant decrease of hemoglobin level is often observed in patients undergoing SAVR, which does not always reflect a condition of major perioperative bleeding. Acute kidney injury (AKI) was defined according to the KDIGO criteria [18], i.e., postoperative increase of creatinine ≥ 1.5 times, increase of creatinine ≥ 26.5 micromol/L or need for renal replacement therapy.

### Statistical analysis

Continuous variables are reported as means and standard deviations and median with interquartile range when indicated. Categorical variables are reported as counts and percentages. Univariate analysis in the unmatched population was performed using the Mann–Whitney, Fisher’s and Chi-square tests. Independent predictors of mild-to-severe PVR were identified by logistic regression analysis with regression models including covariates with *p* < 0.20 in univariate analysis and using a backward stepwise method. A propensity score was estimated using non-parsimonious logistic regression including the following clinical variables: age, gender, body mass index, hemoglobin, estimated glomerular filtration rate according to the Chronic Kidney Disease Epidemiology Collaboration (CKD-EPI) equation, diabetes, stroke, pulmonary disease, atrial fibrillation, extracardiac arteriopathy, New York Heart Association class IV, Geriatric Frailty Status Scale 2–3 [19], urgent/emergency procedure, prior pacemaker, acute heart failure within 60 days from the index procedure, prior cardiac surgery, prior percutaneous coronary intervention, left ventricular ejection fraction ≤ 50%, number of diseased vessels and STS score [20]. One-to-one propensity score matching was performed with the nearest neighbor matching method using a caliper width of 0.01. Standardized differences ≤ 0.10 were considered as an adequate balance between the cohorts. The paired *t*-test, the McNemar test and Stuart–Maxwell test were used to investigate any difference in the early outcomes between propensity score-matched pairs. Differences in late outcomes were evaluated by the Kaplan–Meier method with the log-rank test. *P* < 0.05 was set for statistical significance. All data management and analyses were conducted using Stata v. 15.1 (StataCorp LLC, College Station, Texas, USA) and SPSS v. 25.0 (IBM Corporation, New York, USA) statistical softwares.

## Results

### Clinical characteristics

The FinnValve registry included 6463 patients (4333 SAVR patients and 2130 TAVR patients). BAV was present in 920 out of 4333 (21.2%) SAVR patients. Hundred and three out of 2130 (4.8%) TAVR patients had proven BAV morphology according to the preoperative computed tomography findings. These 1023 patients with BAV were the subjects of the present analysis. Patients with BAV were significantly younger than those with tricuspid aortic valve (71.6 ± 7.6 vs. 78.2 ± 6.6 years, *p* < 0.0001). The proportion of TAVR for stenotic BAV in each center ranged from 2.0% to 8.2%, and 80.6% of TAVR procedures for stenotic BAV were performed after 2014.

The baseline characteristics of the unmatched and matched cohorts are summarized in Table 1. TAVR patients were significantly older and had a significantly higher operative risk compared to SAVR patients. The mean follow-up in this series was 4.6 ± 2.7 years and median 3.0 years (IQR 0.6) (TAVR cohort: mean 2.4 ± 1.4 years, median 2.1 years, IQR 1.6; SAVR cohort: mean 4.9 ± 2.7 years, median 3.0, IQR 0.3, *p* < 0.0001).

**Table 1:** Characteristics and operative data of unmatched and propensity score-matched cohorts

Variables	Unmatched cohorts				Propensity score-matched cohorts			
	TAVR (*n* = 103)	SAVR (*n* = 920)	Standardized difference	*p*-value	TAVR (*n* = 75)	SAVR (*n* = 75)	Standardized difference	*p*-value
*Baseline data*								
Age (years)	77.1 ± 8.1	70.9 ± 7.2	0.807	< 0.0001	75.8 ± 8.4	75.7 ± 6.3	0.009	0.577
Women	43 (41.7)	386 (42.0)	0.004	0.967	33 (44.0)	34 (45.3)	0.027	0.870
Body mass index (kg/m^2^)	27.8 ± 5.1	27.0 ± 4.6	0.170	0.112	27.2 ± 4.6	27.3 ± 4.9	0.026	0.966
Hemoglobin (g/L)	12.9 ± 1.5	13.6 ± 1.4	0.460	< 0.0001	13.1 ± 1.5	13.1 ± 1.5	0.029	0.827
eGFR (ml/min/1.73m^2^)	62 ± 20	74 ± 17	0.607	< 0.0001	65 ± 20	66 ± 198	0.015	0.888
EuroSCORE II (%)	4.8 ± 3.9	3.0 ± 4.2	0.446	< 0.0001	4.0 ± 3.3	3.8 ± 3.6	0.060	0.385
STS score (%)	3.4 ± 2.0	2.2 ± 2.1	0.555	< 0.0001	2.9 ± 1.7	3.1 ± 3.2	0.060	0.334
*Comorbidities*								
Diabetes	28 (27.2)	177 (19.2)	0.189	0.056	16 (21.3)	11 (14.7)	0.174	0.288
Stroke	10 (9.7)	43 (4.7)	0.196	0.029	6 (8.0)	7 (9.3)	0.047	0.772
Pulmonary disease	26 (25.2)	126 (13.7)	0.295	0.002	16 (21.3)	19 (25.3)	0.009	0.562
Extracardiac arteriopathy	10 (9.7	68 (7.4)	0.083	0.401	7 (9.3)	8 (10.7)	0.044	0.785
Atrial fibrillation	50 (48.5)	160 (17.4)	0.702	< 0.0001	29 (38.7)	38 (50.7)	0.243	0.139
Frailty GSS 2–3	12 (11.7)	20 (2.2)	0.380	< 0.0001	5 (6.3)	7 (8.8)	0.095	1.000
Prior cardiac surgery	14 (13.6)	12 (1.3)	0.481	< 0.0001	4 (5.3)	6 (8.0)	0.107	0.513
Prior percutaneous intervention	19 (18.4)	61 (6.6)	0.362	< 0.0001	12 (16.0)	6 (8.0)	0.248	0.208
Prior permanent pacemaker	8 (7.8)	33 (3.6)	0.181	0.040	5 (6.7)	2 (2.7)	0.191	0.442
LVEF			0.269	0.016			0.009	0.998
> 50%	66 (64.7)	706 (76.7)			50 (66.7)	51 (68.0)		
30–50%	31 (30.4)	180 (19.6)			21 (28.4)	21 (28.0)		
< 30%	5 (4.9)	34 (3.7)			3 (4.1)	3 (4.0)		
NYHA classes			0.107	< 0.0001			0.321	0.152
III	75 (72.8)	393 (42.7)			52 (69.3)	41 (54.7)		
IV	6 (5.8)	79 (8.6)			4 (5.3)	4 (5.3)		
AHF/critical preoperative state	8 (7.8)	106 (11.5)	0.127	0.251	7 (9.3)	8 (10.7)	0.044	0.785
Coronary artery disease	26 (25.2)	286 (31.1)	0.130	0.222	21 (28.0)	21 (28.0)	0.000	1.000
No. of diseased vessels			0.365	0.028			0.154	0.641
1-vessel disease	19 (18.4)	150 (16.3)			14 (18.7)	11 (14.7)		
2-vessel disease	7 (6.8)	83 (9.0)			7 (9.3)	10 (13.3)		
3-vessel disease	0	53 (5.8)			0	0		
*Procedural data*								
Urgent procedure	6 (5.8)	109 (11.8)	0.213	0.067	6 (8.0)	4 (5.3)	0.107	0.513
Coronary revascularization	6 (5.8)	257 (27.9)	0.617	< 0.0001	5 (6.7)	19 (25.3)	0.526	< 0.0001
Transapical access	6 (5.8)	–	–	–	5 (6.7)	0	–	–

*AHF *acute heart failure event within 60 days, *eGFR* estimated glomerular filtration rate, *EuroSCORE * European system for cardiac operative risk evaluation, *GSS *geriatric status scale, *LVEF *left ventricular ejection fraction, *NYHA *New York Heart Association, *SAVR* surgical aortic valve replacement, *SPAP *systolic pulmonary artery pressure, *STS *Society of Thoracic Surgeons, *TAVR *transcatheter aortic valve replacement

Values are number and percentages (in parentheses) or mean ± standard deviation

**Fig. 1:**
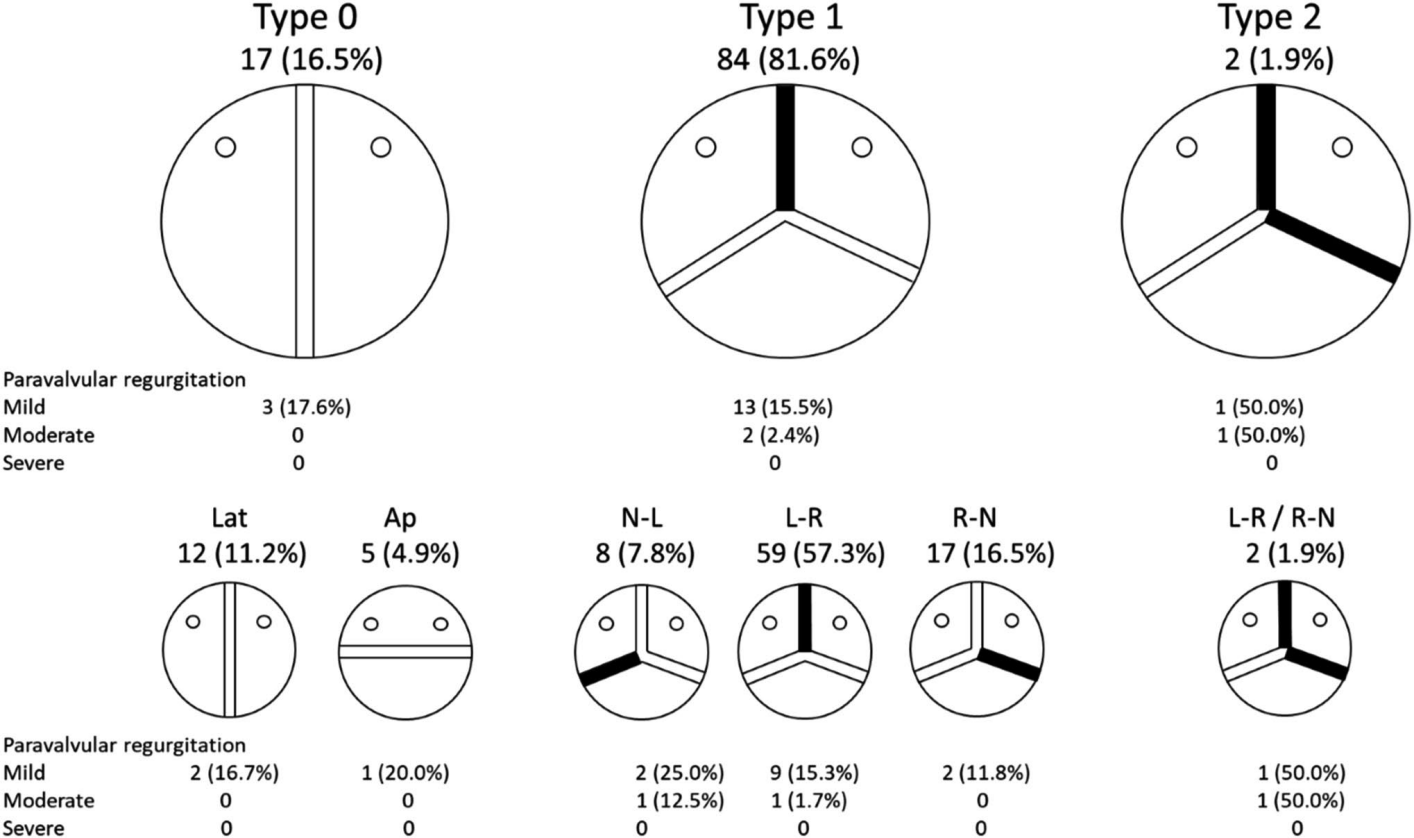
Morphological patterns of BAV of patients who underwent TAVR classified according to the Sievers–Schmidtke’s criteria. Values are prevalences of morphological patterns and their related incidences of paravalvular regurgitation

The morphology of BAVs of TAVR patients according to the Sievers–Schmidtke’s criteria is summarized in Fig. 1. The BAV type 1 morphology was the most frequent (81.6%) followed by the type 0 (16.5%) and the type 2 (1.9%) (Fig. 1).

### Outcomes in the unmatched cohorts

In the unmatched cohorts, TAVR had similar 30-day mortality (0.9% vs. 2.4%, *p* = 0.721), but significantly higher 2-year mortality (11.0% vs. 6.5%, *p* < 0.0001) than SAVR. Device success after TAVR was comparable to SAVR (94.2% vs. 97.1%, *p* = 0.115). Prothesis migration (1.9% vs. 0%, p = 0.010), major vascular complications (5.8% vs 1.3%, *p* < 0.0001), mild-to-severe PVR (19.4% vs. 7.9%, *p* < 0.0001) and new permanent pacemaker implantation (11.3% vs. 4.8%, *p* = 0.006) were significantly more frequent after TAVR compared to SAVR. Moderate-to-severe PVR (2.6% vs. 0.7%, *p* = 0.067) also tended to be more frequent after TAVR. SAVR was associated with higher rate of blood transfusion (60.1% vs 14.9%, *p* < 0.0001), severe bleeding (E-CABG bleeding grades 2–3, 17.4% vs. 4.0%, *p* < 0.0001), AKI (13.2% vs. 4.0%, *p* = 0.006), and longer hospital stay (8.0 ± 6.0 vs. 4.4 ± 3.1 days, *p* < 0.0001). The other adverse events were equally distributed in the unmatched cohorts (Table 2).

**Table 2:** Early postoperative adverse events

	Unmatched cohorts			Propensity score-matched cohorts		
Variable	TAVR (*n* = 103)	SAVR (*n* = 920)	*p*-value	TAVR (*n* = 75)	SAVR (*n* = 75)	*p*-value
30-day death	1 (0.9)	22 (2.4)	0.721	1 (1.3)	4 (5.3)	0.375
Device success	97 (94.2)	893 (97.1)	0.115	72 (96.0)	70 (93.3)	0.727
Stroke	3 (2.9)	32 (3.5)	1.000	3 (4.0)	6 (8.0)	0.508
Conversion to cardiac surgery	0	–	–	0	–	–
Prosthesis migration	2 (1.9)	0	0.010	2 (2.7)	0	0.500
Deep sternal wound infection/mediastinitis	0	14 (1.5)	0.384	0	2 (2.7)	0.500
Coronary ostium occlusion	1 (1.0)	2 (0.2)	0.273	1 (1.3)	0	1.000
Annulus rupture	0	0	–	0	0	–
Aortic dissection/rupture	1 (1.0)	6 (0.7)	0.525	0	1 (1.3)	1.000
Major vascular complication	6 (5.8)	12 (1.3)	< 0.0001	4 (5.3)	1 (1.3)	0.375
RBC transfusion	14 (14.9)	549 (60.1)	< 0.0001	9 (12.3)	52 (69.3)	< 0.0001
RBC transfusion (units)	0.4 ± 1.0	2.5 ± 4.5	< 0.0001	0.3 ± 0.8	2.7 ± 2.8	< 0.0001
RBC transfusion > 4 units	2 (2.0)	132 (15.0)	< 0.0001	1 (1.4)	13 (17.3)	< 0.0001
Reoperation for bleeding	3 (2.9)	68 (7.4)	0.102	2 (2.7)	9 (12.0)	0.065
E-CABG bleeding grades 2–3	4 (4.0)	159 (17.4)	< 0.0001	2 (2.7)	16 (21.3)	0.001
Acute kidney injury	4 (4.0)	120 (13.2)	0.006	2 (2.7)	12 (16.2)	0.008
New renal replacement therapy	2 (1.9)	15 (1.6)	0.686	1 (1.3)	2 (2.7)	1.000
Paravalvular regurgitation	20 (19.4)	73 (7.9)	< 0.0001	12 (16.0)	8 (10.7)	0.454
Mild	17 (16.5)	67 (7.3)		12 (16.0)	6 (8.0)	1.000
Moderate	3 (2.9)	1 (0.1)		0	0	
Severe	0 (0)	5 (0.5)		0	2 (2.7)	
Atrial fibrillation	47 (45.6)	464 (50.4)	0.355	31 (41.3)	53 (70.7)	< 0.0001
New permanent pacemaker	11 (11.6)	43 (4.8)	0.006	8 (11.4)	4 (5.5)	0.388
Hospital stay (days)	4.4 ± 3.1	8.0 ± 6.0	< 0.0001	4.4 ± 3.0	8.1 ± 4.4	< 0.0001

*E-CABG *European coronary artery bypass grafting, *RBC *red blood cell, *SAVR *surgical aortic valve replacement, *TAVR *transcatheter aortic valve replacement, *VARC *Valve Academic Research Consortium

^a^it includes also intervention for peripheral bleeding

Values are number and percentages (in parentheses) or mean ± standard deviation

Figure 1 summarizes the incidence of mild-to-severe PVR after TAVR according to different BAV morphologies. Mild-to-severe PVR was higher in type 2 BAV compared to other types of BAVs (type 0 17.6%, type 1 17.9%, type 2 100%, adjusted for new-generation prostheses, *p* = 0.823), but the difference did not reach statistical significance. The incidence of mild-to-severe PVR was significantly different according to the subtypes of BAV (Lat 16.7%, Ap 20.0%, N-L 37.5%, L-R 16.9%, R-N 11.8%, L-R/R-N 100%, adjusted for newer-generation prostheses, *p* = 0.025).

Table 3 summarizes the incidence of PVR of different severity according to the implanted prostheses. Among TAVR patients, the risk of mild-to-severe PVR was lower with newer devices compared to older ones (11.9% vs. 52.6%, *p* < 0.0001) (Table 3). When newer-generation TAVR devices were evaluated, the rates of mild-to-severe PVR (11.9% vs. 7.9%, *p* = 0.223) and moderate-to-severe PVR (0% vs. 0.8%, *p* = 1.000) were comparable to SAVR.

**Table 3:** Type of valve prostheses and incidence of paravalvular regurgitation

Prosthesis	No. of patients	Mild regurgitation	Moderate regurgitation	Severe regurgitation
*TAVR*				
CoreValve	2	1 (50.0)	0	0
CoreValve Evolut	8	2 (25.0)	0	0
Lotus	11	0	0	0
Sapien 3	63	8 (12.7)	0	0
Sapien XT	17	6 (35.3)	3 (17.6)	0
Symetis Acurate Neo	2	0	0	0
SAVR*				
3F Enable	1	0	0	0
Epic	132	8 (6.1)	0	0
Freedom SOLO	6	2 (33.3)	0	0
Hancock II	54	2 (3.7)	0	0
Inspiris Resilia	3	0	0	0
Intuity Elite	3	1 (3.3)	0	0
Mitroflow/Crown	138	13 (3.4)	0	1 (0.7)
Mosaic	20	1 (5.0)	0	0
Perceval	8	1 (12.5)	0	0
Perimount Magna Ease	314	20 (6.4)	1 (0.3)	2 (0.6)
Soprano	36	2 (5.6)	0	1 (2.8)
Trifecta	204	17 (8.3)	0	1 (0.5)

*SAVR *surgical aortic valve replacement, *TAVR *transcatheter aortic valve replacement

Values are number and percentages (in parentheses)

^*^Missing data in one patient

Among TAVR patients, the type of prosthesis was the only independent predictor of mild-to-severe PVR (*p* = 0.040). The incidences of PVR according to different prostheses are summarized in Table 3. Self-expandable prostheses were not associated with lower risk of PVR compared to mechanically/balloon expandable prostheses (*p* = 0.698).

Among SAVR patients, extracardiac arteriopathy was the only independent predictor of mild-to-severe PVR (*p* = 0.007, odds ratio 2.628, 95% confidence interval 1.304–5.297).

The risk of new permanent pacemaker implantation (14.5% vs. 4.8%, *p* = 0.006) was significantly higher than SAVR also with newer-generation TAVR prostheses.

Two-year rates of repeat operation for any aortic valve prosthesis-related complication was 0% after TAVR and 1.3% after SAVR (*p* = 0.249).

### Outcomes in propensity score-matched cohorts

Propensity score matching resulted in 75 pairs of patients with balanced baseline variables and comparable operative risk (Table 1). A few variables had marginally high standardized differences (Table 1). As expected, concomitant coronary revascularization was more frequent in the SAVR cohort despite a comparable prevalence of coronary artery disease and number of diseased vessels between the matched cohorts.

**Fig. 2:**
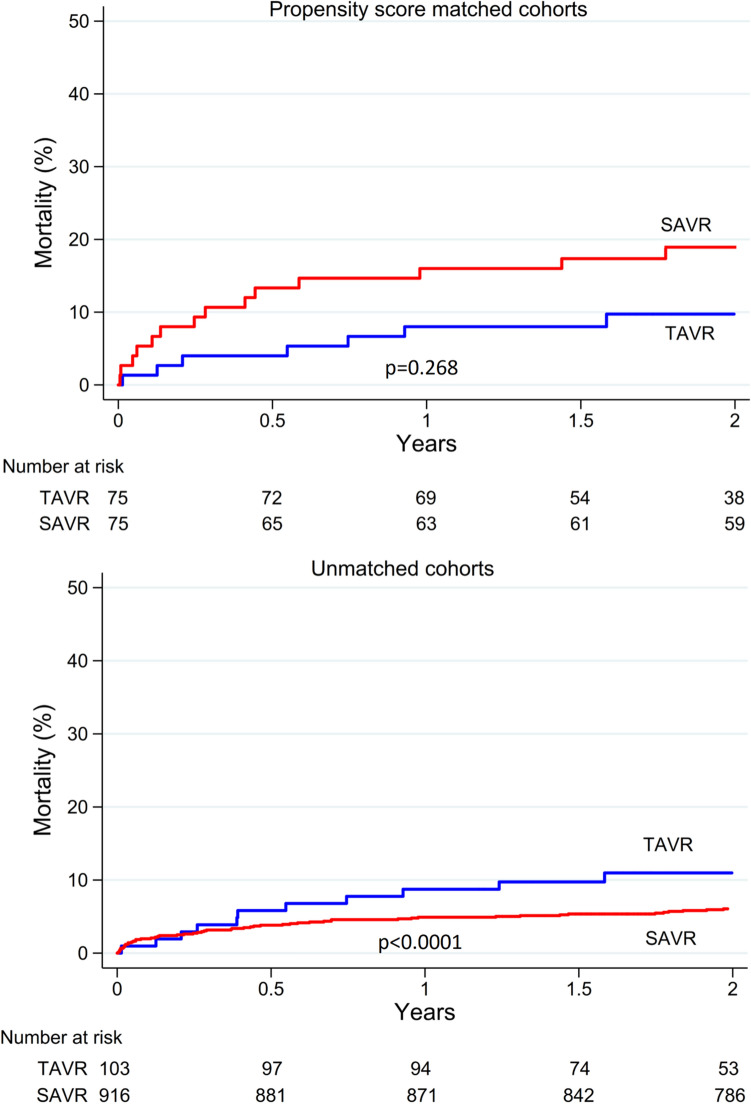
Kaplan–Meier estimates of all-cause mortality of unmatched cohorts and of propensity score-matched cohorts of patients with stenotic bicuspid aortic valve who underwent transcatheter (TAVR) or surgical aortic valve replacement (SAVR)

Thirty-day mortality was 1.3% after TAVR and 5.3% after SAVR, but the difference did not reach statistical significance (*p* = 0.375). Two-year mortality was comparable between TAVR and SAVR (9.7% vs. 18.7%, Log-rank test *p *= 0.268) (Fig. 2).

Device success was numerically higher after TAVR (96.0% vs. 93.3%, *p* = 0.727) compared to SAVR, but the difference was not statistically significant (Table 2). SAVR was associated with increased rates of blood transfusion, severe bleeding, AKI, atrial fibrillation and prolonged hospital stay. Other adverse events were equally distributed between the matched cohorts (Table 2). In these propensity score-matched cohorts, TAVR was associated with mild-to-severe PVR (16.0% vs. 10.7%, p = 0.454) and moderate-to-severe PVR (0% vs. 2.7%, *p* = 0.500) comparable to SAVR.

Two-year rates of repeat operation for any aortic valve prosthesis-related complication were 0% after TAVR and 1.3% after SAVR (Log-rank test *p* = 0.317).

Propensity score matching among patients without coronary artery disease resulted in 50 pairs in whom TAVR tended to have lower 30-day (TAVR 0% vs. SAVR 10.0%, McNemar test *p* = 0.063) and comparable 2-year mortality (TAVR 10.4% vs. SAVR 14.0%, Log-rank test *p* = 0.789).

## Discussion

This study provides comparative data on TAVR versus SAVR in patients with stenotic BAV from an unselected nationwide registry and its main findings are: (1) the prevalence of BAV was rather high (16%) among elderly undergoing TAVR or SAVR for AS; (2) patients with BAV were significantly younger than those with tricuspid AS; (3) the majority of TAVR for stenotic BAV were performed during the last years of the study period; (4) when adjusted for baseline covariates, TAVR had early and mid-term mortality comparable to SAVR; (5) TAVR, particularly when newer devices were used, was associated with rates of device success and PVR comparable to SAVR; (6) PVR after TAVR may differ according to BAV morphology.

The present findings have significant clinical implications because patients with BAV are exposed to accelerated aortic valve calcification and require invasive treatment at younger age than patients with tricuspid aortic valve. This means that BAV patients are expected to have a long-life expectancy after intervention and TAVR in these patients should be performed only when high device success, low rate of PVR and prosthesis durability are guaranteed. These issues explain why the prevalence of BAV in TAVR cohorts were much lower than in the SAVR cohorts and ranged from 3.3% to 5.4% in two recent studies [14, 21], which are comparable to the prevalence of BAV in the present TAVR cohort (4.8%).

The largest randomized trial for TAVR in high-risk AS patients extends 5 years and several recent trials demonstrated excellent results in low- and intermediate risk patients with tricuspid aortic valve [5, 6, 22]. Treatment of BAV with TAVR has initially been classified as off-label, but increasing operator experience and new transcatheter technology has encouraged its use in these patients [23–25]. Indeed, in our study, 80% of TAVR patients were treated after 2014 employing newer-generation devices. There was no difference in all-cause 30-day and 2-year mortality between TAVR- and SAVR-matched cohorts of our study. Previously, TAVR has been shown to achieve similar outcomes in stenotic BAV and tricuspid aortic valves [8, 24]. Makkar et al. [14] demonstrated in 2691 propensity score-matched pairs from the Society of Thoracic Surgeons (STS)/American College of Cardiology (ACC) Transcatheter Valve Therapies Registry comparable 30-day and 1-year survival with TAVR in patients with BAV and tricuspid aortic valve. Similar results were reported from the same registry by Forrest et al. [21] among patients treated with self-expandable Evolut R or Evolut PRO prostheses.

The incidence of PVR after TAVR has significantly decreased from the early days of transcatheter practice, and moderate-to-severe PVR ranged from 0.6% to 3.6% in the most recent studies [26, 27]. Asymmetrical annulus and calcified raphes may contribute to increased risk of PVR in BAV patients [28]. Still, Halim et al. [10] compared TAVR for stenotic BAV and tricuspid aortic valve using mostly newer TAVR devices from the STS database. They reported mild PVR in 26.7% of patients, moderate in 4.1% and severe in 0.3% of BAV group patients, with no significant difference compared to tricuspid aortic valves [10]. A large multicenter study [14] showed that moderate-to-severe PVR was observed in 1.5% of patients after TAVR with the newer-generation Sapien 3 prosthesis. When newer-generation TAVR devices were assessed in the present study, the rate of mild PVR was 11.9% and no moderate or severe PVR was observed. These results were comparable to SAVR.

Herein, we observed that the prevalence of different BAV morphologies in patients undergoing TAVR is comparable to those of patients undergoing surgery for aortic valve diseases reported by Sievers and Schimdtke [15]. Although the small size of this study might introduce bias, we observed that BAVs with type 1 N-L and type 2 L-R/R-N morphologies had a significantly higher incidence of mild-to-severe PVR (37.5% and 100%, respectively) compared to other types of BAVs. Therefore, further studies investigating BAV morphology in TAVR patients are needed, because, if the present findings are confirmed, these subtypes of BAVs may contraindicate TAVR.

Annular and/or aortic root enlargement are/is often present in BAV, and these patients are generally treated with SAVR because of the need for concomitant aortic procedure or lack of appropriate size TAVR prostheses [29]. However, the FinnValve registry included only patients who underwent aortic valve replacement with or without coronary revascularization; therefore, patients with significant dilatation of the aortic root/ascending aorta were not included in this registry. BAV with associated aortic dilatation has been suggested to increase the risk of aortic complications during TAVR as aortic root injury has occurred in up to 4.5% of BAV with early generation TAVR devices [22]. This finding was not confirmed in our study as no annulus rupture occurred after TAVR. Low or abnormally located coronary ostia is also a matter of concern in these patients [30], but in our series, this complication occurred only in one patient (0.9%) after TAVR. Excessive calcification is often present in stenotic BAV and may affect device success in TAVR [30]. In this study, the device success was rather high, but we cannot exclude a selection bias as patients with extremely calcified BAV leaflets and annulus might have been preferentially treated with SAVR. The risk of stroke in TAVR has been postulated to fall below the risk of stroke in SAVR; however, BAV may increase its risk [5, 14]. Makkar et al. [14] reported an increased hospital stroke rate (2.1% vs. 1.2%, *p* = 0.01) in patients with BAV compared to those with tricuspid aortic valve after TAVR. In our unmatched cohorts, the rate of hospital stroke was 2.9% after TAVR and 3.5% after SAVR (*p* = 0.500), whilst in the propensity score-matched cohorts was 4.0% after TAVR and 8.0% after SAVR (*p* = 0.508). In the FinnValve registry, the hospital stroke rate was not significantly lower than either TAVR (2.5%, *p* = 1.000) or SAVR (3.9%, *p* = 0.556) for stenotic tricuspid aortic valve.

### Limitations

The retrospective nature is the main limitation of this study. Second, the selection of treatment method was made by the Heart Teams based on current best knowledge and patient’s conditions and might have been affected by unmeasured confounders. The differences in baseline characteristics between the study cohorts were adjusted using propensity score matching, but lack of randomization might introduce significant bias. Third, we do not have data on the phenotypes of BAV in SAVR patients, which might have had an impact on the decision-making process and clinical results. Fourth, we defined device success based only on 30-day mortality, correct positioning of a single prosthetic heart valve into the proper anatomical location, and no moderate or severe PVR. This was due to the lack of data on postoperative transvalvular gradient in our registry. Fifth, data on postprocedural intra-valvular regurgitation were not collected in this registry and this prevented an analysis of this event. Last, the relatively small size of the TAVR cohort might be a source of type II error in the present analysis.

## Conclusions

In patients with stenotic BAV, 30-day and 2-year mortality as well as device success and PVR were comparable after TAVR and SAVR. Newer-generation devices were associated with a reduced risk of PVR after TAVR in BAV patients. We observed that BAVs with type 1 N-L and type 2, L-R/R-N BAV morphologies had an excessive risk of mild-to-severe PVR. These findings should be confirmed in larger studies, because these BAV morphologies may contraindicate TAVR.

## Data Availability

Not permitted.

## Abbreviations

AHF: Acute heart failure
AKI: Acute kidney injury
AS: Aortic stenosis
BAV: Bicuspid aortic valve
CKD-EPI: Chronic kidney disease epidemiology collaboration
E-CABG: European coronary artery bypass grafting registry
eGFR: Estimated glomerular filtration rate
EuroSCORE: European system for cardiac operative risk evaluation
GSS: Geriatric status scale
KDIGO: Kidney disease improving global outcomes
LVEF: Left ventricular ejection fraction
NYHA: New York Heart Association
PVR: Paravalvular regurgitation
RBC: Red blood cell
SAVR: Surgical aortic valve replacement
SPAP: Systolic pulmonary artery pressure
STS: Society of Thoracic Surgeons
TAVR: Transcatheter aortic valve replacement
VARC-2: Valve Academic Research Consortium 2
